# Facilitating Utilization of Evidence-Informed Management by Nurse Managers in Healthcare Facilities: An Integrative Literature Review

**DOI:** 10.1155/2024/6649401

**Published:** 2024-05-21

**Authors:** Diane Roobasoundhrie Chetty, Wilma ten Ham-Baloyi, Dalena R. M. van Rooyen, Allison Herlene Du Plessis, Joanne Naidoo

**Affiliations:** ^1^Department of Nursing Science, Faculty of Health Sciences, Nelson Mandela University, Gqeberha, South Africa; ^2^Faculty of Health Sciences, Nelson Mandela University, Gqeberha, South Africa

## Abstract

**Methods:**

An integrative review of the literature was conducted, including peer-reviewed articles published between 2010 and 2022. The databases used were BioMed Central, CINAHL Complete, MEDLINE Complete, PubMed (via EBSCOhost), the Complimentary Index (Taylor and Francis, Elsevier, Wiley, and Springer), Sabinet, ScienceDirect, and Scopus, followed by a manual search using Google Scholar and a citation search. Johns Hopkins Nursing Evidence-based Practice Research and non-Research evidence tools were used for appraisal. Thematic analysis was used to synthesize the extracted data.

**Results:**

Based on thirteen determinants influencing nurse managers' utilization of evidence-informed management practices, three themes were identified from a total of thirteen relevant studies: (1) Nurse manager determinants in utilization of evidence-informed management (Microlevel); (2) Organizational determinants in utilization of evidence-informed management (Mesolevel); (3) External stakeholders and context determinants of utilization of evidence-informed management practices (Macrolevel).

**Conclusion:**

The themes were found to be interconnected and interdependent, facilitating the effective utilization of evidence-informed management by nurse managers at micro-, meso-, and macrolevels, but highlight the need for strengthening health systems and support. Future studies are required to provide a more comprehensive understanding of the determinants influencing nurse managers' utilization of evidence-informed management practices. *Implications for Nursing Management*. For nurse managers to optimally utilize evidence-informed management, executive management and policymakers require to provide resources and support such as continuous education, incentives, effective communication, funding structures, and ownership.

## 1. Introduction

Evidence-informed management, also known as evidence-based management, is a prominent approach in management and public health [1, 2]. It involves systematically utilizing the best available evidence to inform decision making, improve management practices in patient care and nursing staff supervision, and achieve desirable organizational outcomes [3, 4]. “Evidence” encompasses data from research and non-research sources such as professional experience, feedback, and organizational information [5]. While “evidence-based management” tends to emphasize empirical evidence, “evidence-informed management” allows greater flexibility [6, 7]. Nurse managers, or middle managers, play a crucial role in initiating, guiding, and sustaining evidence-informed management practices [8–10]. Utilizing evidence-informed management leads to reduced bias, improved efficiency, better patient outcomes, and strategic planning support [9–11]. Failure to employ evidence-informed management can result in ineffective decision making, increased costs, reduced productivity, and negative effects on patient satisfaction and organizational reputation [12].

Enhancing the utilization of evidence-informed management practices among nurse managers is crucial for optimizing clinical and organizational advantages [13]. Understanding the facilitators in complex healthcare environments is essential [1], yet its application in nurse management remains novel [10]. Additionally, there is a scarcity of empirical information on evidence-informed management in relation to nurse managers, posing limitations to understanding its uptake and utilization [14, 15], potentially leading to ineffective practices and policy implementation [12, 16].

Moreover, a gap exists in integrative literature reviews conducted in English on this topic, underscoring the need for a comprehensive review [17]. Such a review could offer insights into strategies and interventions for nurse managers to utilize evidence-informed management effectively, thereby enhancing management practices and improving nursing and patient outcomes.

Thus, the objective of this review is to summarize best available evidence on facilitating utilization of evidence-informed management by nurse managers in healthcare facilities. The review question guiding the review was: “What is the best evidence available regarding the facilitation of utilizing evidence-informed management by nurse managers in healthcare facilities?”

## 2. Materials and Methods

The review was conducted according to Whittemore and Knafl's [18] stages for an intergrative literature review. These stages include problem identification, searching, selecting, appraising, analysing and synthesizing, and presenting the data.

### 2.1. Search and Selection

**Figure 1:**
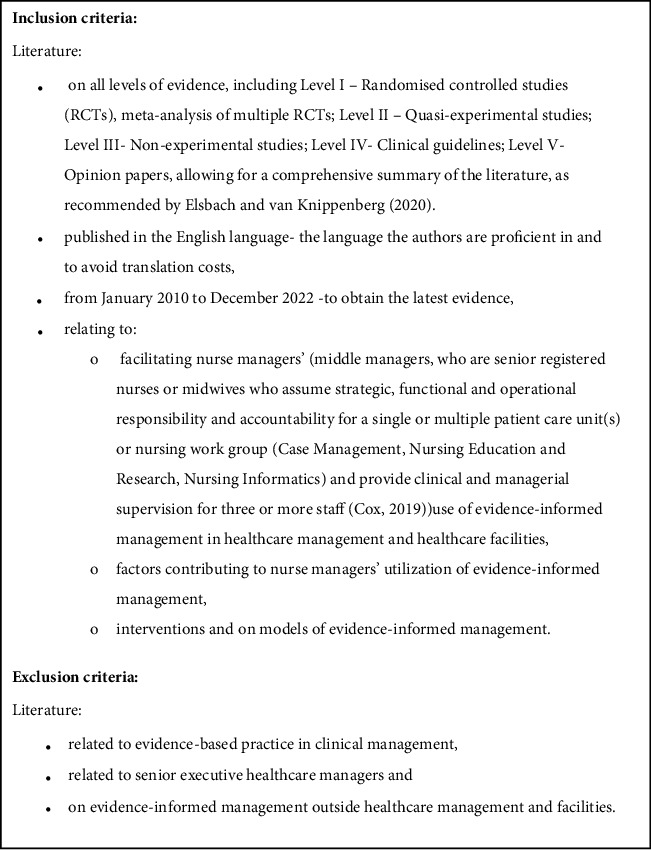
Eligibility criteria.

With the assistance of the faculty librarian, a comprehensive search was conducted in November and December 2022. The following electronic databases were searched: BioMed Central, as well as CINAHL Complete, MEDLINE Complete, and PubMed (via EBSCOhost), the Complimentary Index (Taylor and Francis, Elsevier, Wiley, and Springer), Sabinet, ScienceDirect, and Scopus. Thereafter, a manual search for grey literature using Google Scholar and a citation search were completed. Databases were chosen in consultation with the faculty librarian, adhering to recommendations by Bramer et al. [19] for systematic review searches, ensuring retrieval of the most relevant scientific data. The following key search terms and/or phrases were used: “evidence-informed management” OR “evidence-based management” AND “Nurse Manager” AND “utilization” OR “facilitation” AND “healthcare” OR “health facility.” Boolean operators assisted in further filtering the search. Key search terms were selected in consultation with the faculty librarian, in accordance with the PICO elements. The PICO elements included P (opulation): nurse manager, I (ntervention): evidence-informed management, C (ontext): health-care facility or context, and O (utcome): facilitating the utilization of the intervention (evidence-informed management), focussing on the “process” as an outcome as compared to improved management practices, nursing and patient outcomes. The Boolean operator “OR” and “AND” was used to combine these search terms with different syntaxes adapted to each database. Screening was done according to the following eligibility criteria as outlined in Figure 1.

After deduplication, titles and abstracts of all documents and full texts obtained were screened for relevance by D.R.C. and J.N. independently, using the inclusion and exclusion criteria. The researcher initially exported records to EndNote version X9 and thereafter recorded and tracked articles in Microsoft Excel. Each step used in the searching and selecting processes is illustrated in the Preferred Reporting Items for Systematic Reviews and Meta-Analyses (PRISMA) flow diagram [20].

#### 2.1.1. Critical Appraisal

After data reduction through the screening for relevance was finalized, critical appraisal using the Johns Hopkins Nursing Evidence-based Practice Research (for studies with Level I, II, and III evidence) and non-Research evidence appraisal tools (for literature with Level IV and V evidence) was undertaken by D.R.C. and J.N. independently. These tools are generic tools that present a structured approach to appraising studies that present evidence of different strengths and quality using a widely established quality grading scale of high, good, and low quality [21]. D.R.C. created an Excel spreadsheet and compiled a list of the articles for review, which was sent to the independent reviewer (J.N.) with an electronic fillable Word document of the applicable appraisal tools. For studies with Level I, II, and III evidence, the items related to the strength of the study design, study results, and study conclusions were answered by clicking “yes” or “no” and comments were made on the pertinent study findings and recommendations. For literature with Level IV and V evidence, similarly, the items pertaining to the type of evidence were completed by clicking “yes” or “no” and comments were made on the pertinent study findings and recommendations. In addition, literature was graded. Only literature that met the *High* (A quality evidence) and *Good* (B quality evidence) quality grading scale according to Dang and Dearholt [21] was included for data extraction. High quality evidence included studies with rigorous designs that produced consistent, generalizable results (quantitative) or those studied that included measures of trustworthiness (qualitative) or were scientific/expert opinion papers or literature reviews. Good quality evidence included studies with reasonable designs and results (quantitative), and those that included some measures of trustworthiness (qualitative) or were credible expertise papers (opinion papers and literature reviews) [21]. A briefing meeting was held between D.R.C., J.N., and W.T.H.B. to discuss the compiled list of articles for review, the appraisal tools, timelines for review completion, follow-up, and consensus meetings where critical appraisal outcomes (number of “yes” or “no” answers, and grading) were compared between reviewers and discussed—second and third meetings. W.T.H.B.'s role was to overview the process of critical appraisal and act as consultant when there were discrepancies between the two reviewers.

#### 2.1.2. Data Synthesis

After the critical appraisal, the data from included articles were manually extracted by D.R.C. using a self-developed data extraction tool (Excel spreadsheet), as suggested by Lubbe et al. [22]. The data extraction tool included the reference, aim and setting of the study and summary of the study findings, recommendations, and implication(s) for practice (see supplementary file (available here) for the data extraction table). D.R.C. and J.N. independently utilized a content analysis approach to analyse the data. This approach included reading and re-reading the extracted summarized findings and recommendations as well as the implication(s) for practice from the spreadsheet, assigning codes using an additional column, which were grouped in terms of commonalities (e.g., the phrase “Belief in the evidence is important for evidence-informed management implementation” was grouped under the code “Nurse managers' attitude and behaviour towards evidence-informed management”) [18, 22]. The final code book was derived through verification of both sets of grouped codes. Grouped codes were synthesized by re-categorization under broad headings, with the aim of deriving themes (three themes outlining the various determinants). For example, the grouped code “Nurse managers' attitude and behaviour towards evidence-informed management” was categorized as one of the nurse manager determinants in utilization of evidence-informed management—Theme One. Any disagreements were resolved through consensus discussions between the authors.

### 2.2. Data Presentation

The developed themes were presented in a sequential order, starting with those that had the most supporting evidence and concluding with themes that had comparatively less supportive evidence. The level or strength of the evidence was also described for each theme. Data were reported as a narrative, supported by tables and figures.

### 2.3. Rigor of the Review

Overall quality and rigor of the review process were maintained using the structured process and steps articulated by Whittemore and Knafl [18]. Several steps were taken to mitigate bias and ensure rigor during the review process. In terms of the search, this was conducted by a team of researchers, including an experienced librarian; a thorough search strategy was used with carefully selected keywords and databases and the search was recorded using the PRISMA, as recommended by Batten and Brackett [23]. For the screening and data extraction processes, two reviewers independently screened the literature for inclusion in the study. Furthermore, the use of credible critical appraisal tools, using an independent reviewer, and use of a data extraction form in the form of a spreadsheet enhanced rigor [24, 25]. Finally, the transparent documentation of each step of the review process enhanced replicability of the process [26].

## 3. Results

### 3.1. Search and Selection Results

**Figure 2:**
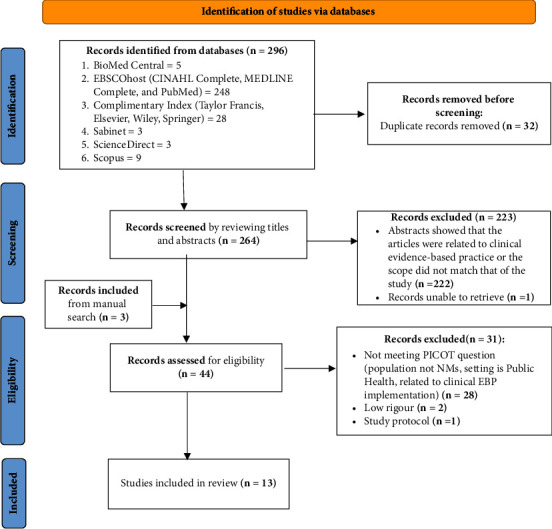
Flow diagram to illustrate data evaluation and reduction (adapted after [20]).

A total of two hundred and ninety-six (*n* = 296) articles were identified from searching the electronic databases. After deduplication and screening titles and abstracts, *n* = 41 articles were retained. The manual search and citation search yielded an additional three articles. A total of *n* = 44 full-text articles were screened for possible inclusion in the selection, leading to the exclusion of *n* = 29 articles and a further exclusion of two (*n* = 2) articles after critical appraisal. Thirteen articles met the inclusion criteria and were therefore included in the review (see Figure 2).

### 3.2. Quality of Evidence

Most studies (*n* = 11) were non-experimental research, including qualitative (*n* = 6), quantitative (*n* = 4), and mixed method (*n* = 1) designs, offering Level III evidence with *Good* (B) quality rating. The remaining two studies (*n* = 2) were non-research studies with Level V evidence and a quality rating of *Good* (B) [21]. The level of evidence and quality rating for all themes and related determinants were III(B), V(B).

### 3.3. Country

The studies were conducted in different countries, namely, Iran (*n* = 4), the United States of America (*n* = 3), Canada (*n* = 2), Australia (*n* = 1), Lebanon (*n* = 1), Pan-European (*n* = 1), and Scotland (*n* = 1).

### 3.4. Healthcare Context

Given the variety of countries in which the studies were conducted, the review of the healthcare contexts indicated diverse multicultural settings with varying socioeconomic and facility ownership policies. Contexts included rural, regional, and urban hospitals.

### 3.5. Determinants and Themes

Following the synthesis, thirteen (*n* = 13) determinants that were reported as facilitators and barriers to nurse managers' evidence-informed management utilization were identified. These determinants pointed to influences at different levels of the healthcare system—that is, the micro- (nurse manager level), meso- (organization level), and macro- (external) levels. The determinants were categorized under three main themes (see Table 1).

A comprehensive discussion of the themes and subthemes is presented in the following section.

#### 3.5.1. Theme One: Nurse Manager Determinants in Utilization of Evidence-Informed Management (Microlevel)

**Figure 3:**
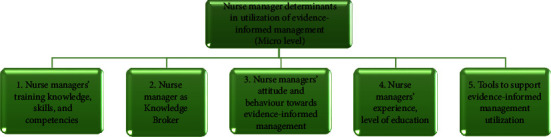
Nurse manager determinants (theme one).

At the individual nurse manager level within the healthcare system, there is a significant expectation and responsibility to effectively use evidence in management practice [13, 17]. Thirteen studies identified five determinants for nurse managers' utilization of evidence-informed management: appropriate training and competencies (*n* = 13), acting as knowledge brokers (*n* = 12), attitudes and behaviours (*n* = 11), experience and education level (*n* = 10), and availability of supportive tools (*n* = 7) (see Figure 3).

#### 3.5.2. Determinant 1: Nurse Managers' Training, Knowledge, Skills, and Competencies

To enhance the utilization of evidence-informed management by nurse managers, ongoing education in skills such as evidence searching, appraisal, interpretation, and application is essential. This education should complement formal training through workshops and reading reports. Creating a supportive learning environment, fostered by peer coaching, mentoring, and exposure to evidence-utilization situations, further enhances nurse managers' capacity [1, 2, 13, 17, 27–32, 34].

#### 3.5.3. Determinant 2: Nurse Manager as Knowledge Broker

Nurse managers, as middle managers, play a pivotal role as information hubs within healthcare settings, bridging the gap between various stakeholders by gathering, translating, and disseminating information. This knowledge brokering function facilitates change and enhances the integration of knowledge into practice. Achieving this role effectively necessitates flattened hierarchical structures, empowering nurse managers with the autonomy and authority to make informed decisions [34, 35].

#### 3.5.4. Determinant 3: Nurse Managers' Attitude and Behaviour towards Evidence-Informed Management

Nurse managers demonstrate positive attitudes and behaviours towards evidence-informed management by showing enthusiasm, motivation, and openness to innovation, along with utilizing performance data to inform decisions. Improving these attitudes and behaviours can be achieved through incentives, adequate resource provision, funding for innovation, and effective communication from senior management. Establishing policies, facilitating shared governance, and enhancing participation in decision making further support this culture [1, 2, 13, 17, 32–35].

#### 3.5.5. Determinant 4: Nurse Manager Experience and Level of Education

Nurse managers with a Bachelor level of education were reported to be positively associated with promoting principles of quality [28]. However, Hasanpoor et al. [13, 17] found no correlation between level of education and experience of using evidence in management practice.

#### 3.5.6. Determinant 5: Tools to Support Evidence-Informed Management Utilization

The synthesized data indicated five (*n* = 5) tools the nurse manager can use to support evidence-informed management utilization (see Table 2).

#### 3.5.7. Theme Two: Organizational Determinants in Utilization of Evidence-Informed Management (Mesolevel)

**Figure 4:**
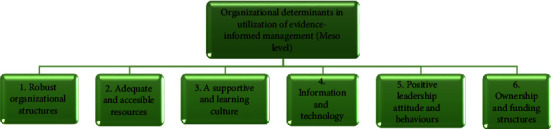
Organizational determinants (theme two).

The 13 included studies identified six organizational determinants (determinants 6–11) influencing evidence-informed management at a mesolevel: (1) robust organizational structures (*n* = 13), (2) adequate and accessible resources (*n* = 13), (3) supportive and learning culture (*n* = 12), (4) information and technology (*n* = 12), (5) positive leadership attitude and behaviours (*n* = 9), and (6) ownership and funding structures (*n* = 4) (Figure 4).

#### 3.5.8. Determinant 6: Robust Organizational Structures

Organizational contexts necessitate the establishment of infrastructure encompassing systems, processes, and structures to carry out the organization's mission in an evidence-informed manner [2, 32]. Robust organizational structures, characterized by a flattened hierarchy and open information flow, facilitate evidence-informed management. These structures, including networking structures for communication and collaboration within and external to the organization, enhance nurse managers' exposure to diverse information sources. They also facilitate engagement in peer-to-peer dialogue with university colleagues, thus strengthening knowledge brokering [13, 17, 31, 33].

#### 3.5.9. Determinant 7: Adequate and Accessible Resources

Evidence-informed management necessitates investment in resources, including research teams, data analysts, and dedicated planning time. Supportive teams and adequate human resources, such as associate nurse managers or clerical support, are crucial to alleviate non-managerial tasks, enabling nurse managers to focus on evidence-informed management [13, 17, 27, 29, 30, 32, 33].

#### 3.5.10. Determinant 8: A Supportive and Learning Culture

A supportive learning culture, essential for evidence-informed management, prioritizes staff development and infrastructure building [2]. This culture necessitates transparent communication on organizational priorities, staff participation, compensation systems, and feedback mechanisms. Accessible education and training programs on performance data usage, along with quality improvement tools, are vital for planning and knowledge translation [13, 17, 31, 32].

#### 3.5.11. Determinant 9: Information and Technology

Organizations can support nurse managers' performance measurement through various resources, such as dashboards and wearable communication devices, aiming to improve efficiency, quality, and patient safety, thus promoting evidence-informed management [12, 13, 17, 30, 32]. Training nurse managers to utilize advanced data interpretation skills, including control and run charts, enhances their ability to utilize available information and technology effectively [12, 13, 17, 32].

#### 3.5.12. Determinant 10: Positive Leadership Attitude and Behaviours

Leaders should embrace suggestions from nurse managers and model evidence-based behaviours, coaching them to do the same and shifting from a crisis management culture [2, 33].

#### 3.5.13. Determinant 11: Ownership and Funding Structures

Ownership of an organization, whether government or private, significantly influences operations at the mesolevel, including the adoption of evidence-informed management. Private for-profit institutions often exhibit greater motivation for evidence use due to financial targets, whereas public institutions may lack such incentives [13, 32]. In low- to middle-income countries, limited resources, inadequate innovation, and a lack of incentivized programs hinder evidence-informed management. These challenges lead to reduced health expenditure, hampered networking, and limited communication and collaboration, particularly in rural areas [27].

#### 3.5.14. Theme Three: The External Stakeholders and Context Determinants of Evidence-Informed Management Utilization (Macrolevel)

**Figure 5:**
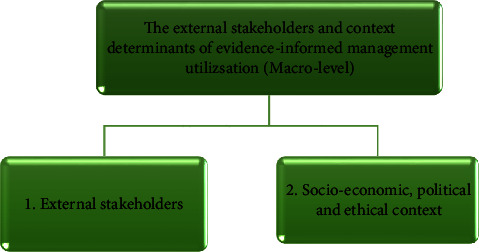
External stakeholders and context determinants (theme three).

At the macrolevel, two determinants (determinants 12 and 13) influencing evidence-informed management utilization were identified: (1) external stakeholders (*n* = 11) and (2) context (socioeconomic, political, and ethical) (*n* = 8) (see Figure 5).

#### 3.5.15. Determinant 12: External Stakeholders

External stakeholders impacting nurse managers' use of evidence-informed management include regulatory bodies mandating performance data submission and setting performance targets [32]. Nurse managers are tasked with measuring this information and achieving the set targets.

#### 3.5.16. Determinant 13: Socioeconomic, Political, and Ethical Context

Strong policies and discourses from the macrolevel external environment, particularly in socioeconomic and political domains, permeate organizational infrastructure at the mesolevel, significantly impacting individual nurse managers' utilization of evidence-informed management at the microlevel. This influence extends through national politics, policy, and socioeconomic reform. Furthermore, ethical influences from government and agency regulations shape access to care and resource allocation through policies and processes [27].

## 4. Discussion

This review aimed to provide a comprehensive synthesis of the most reliable and up-to-date evidence regarding the facilitation of evidence-informed management utilization by nurse managers within healthcare facilities. Through an exhaustive analysis, a total of 13 determinants were identified which significantly influence the utilization of evidence-informed management by nurse managers. These determinants were further categorized into three overarching themes related to the utilization of evidence-informed management: (1) Nurse manager determinants at the microlevel (five determinants); (2) Organizational determinants (six determinants); and (3) External stakeholders and the broader context at the macrolevel (two determinants). It is important to note that despite operating at the microlevel within the organization, nurse managers are influenced by factors at the meso- and macrolevels, emphasizing the significance of considering determinants at all three levels to foster effective utilization of evidence-informed management. Hence, nurse managers should consider the influences from multiple levels to ensure successful utilization of evidence-informed management [37].

At microlevel, the development of nurse managers' knowledge, skills, and competencies in terms of accessing, appraising, and applying evidence in their decision-making processes is essential for effective use of evidence-informed management. This notion is consistent with recommendations made in other studies [38, 39]. Strengthening nurse managers' knowledge, skills, and competencies in evidence-informed management is particularly critical, as a lack of competencies has been reported a common barrier to establishing a culture that supports evidence-informed practices [39, 40]. To enhance nurse managers' competencies, various strategies should be employed and supported by the executive management of a health organization. These include formal education through structured educational programs and ongoing professional development through web-based platforms and online educational resources, all of which should be aimed at enhancing nurse managers' competence and skills in utilizing evidence-informed management in decision-making processes, for example, through training in critical appraisal [39, 41]. Furthermore, access to reliable sources of evidence, such as databases, journals, and research articles, equips nurse managers with the necessary information to make informed decisions while assisting them to gain autonomy to be Knowledge Brokers. In addition, the education pertaining to evidence-based management, whether provided in a formal or informal setting, should also include the utilization of supportive tools identified in our review—including frameworks, models, and decision-making tools.

However, it is important to note that competence in utilizing evidence-informed management may not be sufficient on its own but requires a change in positive attitudes and behaviours. Our review revealed that nurse managers' positive attitudes and behaviours towards evidence-informed management are crucial determinants of utilization, as they encourage them to question prevailing practices. We identified in existing literature several strategies in our review that can enhance nurse managers' attitudes and behaviours towards evidence-informed management, including incentives such as incorporating evidence-informed management competencies into performance evaluation criteria, providing access to adequate human and monetary resources, effective communication that fosters shared governance among nurse managers, and participation in decision-making processes [42]. However, it should be noted that these strategies primarily address extrinsic factors influencing behaviour, while there is limited understanding of the intrinsic factors that drive behaviour towards evidence-informed management utilization, necessitating further exploration in this area. Furthermore, although most nurse manager determinants are largely influenceable by the nurse manager, the findings of our review indicate that the cultivation of an effective Knowledge Broker role (determinant 2) necessitates the presence of flattened hierarchies within organizational structures. Such hierarchical flattening serves as a facilitator for the dissemination and utilization of pertinent evidence. Moreover, this process is not solely contingent upon the influence exerted by nurse managers; rather, it is contingent upon the establishment of a corporate culture characterized by flexibility, egalitarianism, and transparent communication in its approach to evidence-informed management and its paradigms [43].

At mesolevel, our review highlights the significance of organizational determinants in facilitating nurse managers' utilization of evidence-informed management. Organizational determinants are particularly important since they play a crucial role in organizational change and influence evidence-based organizational practices [39, 44]. We identified several key elements of the organizational context that contribute to this utilization, including robust organizational structures, ownership and funding mechanisms, and the availability of adequate and accessible resources, such as information and technology. Furthermore, a supportive and learning culture characterized by positive leadership attitudes and behaviours was found to be crucial, which aligns with findings from other studies [45, 46]. However, it is worth noting that further exploration is needed regarding the determinants concerning the role of ownership and funding structures, as only a limited number of studies supported the relevance of this determinant in the context of nurse managers' utilization of evidence-informed management. Our review has highlighted the impediments posed by resource-constrained environments to the effective utilization of evidence-informed management. However, it is notable that the dearth of studies addressing this specific context within our review limits a comprehensive understanding of the challenges and strategies pertinent to evidence-informed management utilization in such settings. Moreover, the scarcity of innovative and cost-effective interventions aimed at bolstering the adoption of evidence-informed management underscores the necessity for further inquiry and exploration in this domain [47].

At macrolevel, our review revealed that external stakeholders and the broader socioeconomic, political, and ethical context exert indirect effects on nurse managers' utilization of evidence-informed management. For instance, the functioning of a healthcare facility or organization within a larger health system that benefits from a favourable socioeconomic environment, such as sufficient funding, a supportive political landscape with policies promoting innovation and leadership, and an ethical context encompassing appropriate legal systems like auditing and health ombudsman, can facilitate the provision of an enabling organizational context for nurse managers. This finding is consistent with previous research [48] that emphasizes the interplay between the macrolevel context and the capacity-building of nurse managers. Consequently, it is crucial to strengthen healthcare systems by developing the capacity to effectively utilize data to measure the impact of interventions, thereby enhancing the utilization of evidence-informed management. This, in turn, leads to improvements in cost-effectiveness and patient outcomes [49]. Once more, it is important to note that the health system strengthening is not solely contingent upon the actions of nurse managers. Nonetheless, the involvement and input of nurse managers are imperative in facilitating their ability to effectively leverage the utilization of evidence-informed management. Empowering nurse managers with decision-making authority and incorporating their perspectives can contribute significantly to the successful integration of evidence-based approaches within health systems [50]. Thus, while nurse managers may not directly influence the strengthening of health systems, their engagement is essential for promoting the utilization of evidence-informed management.

In conclusion, while our review delineated determinants of evidence-informed management across micro-, meso-, and macrolevels, it is imperative to recognize that these determinants do not operate in isolation. Rather, they interact dynamically, constituting an intricate web of factors that collectively influence the utilization of evidence-informed management practices. This holistic perspective underscores the need for a comprehensive understanding of the interplay among various determinants to effectively foster evidence utilization within organizational contexts.

### 4.1. Limitations

This review has several limitations that should be considered when interpreting the findings. Firstly, the small number of studies related to the facilitation of utilization of evidence-informed management by nurse managers limits in-depth understanding and conclusive evidence on the topic. Secondly, the search for relevant literature was restricted to institutional databases accessible to D.R.C., which may have led to the exclusion of potentially valuable studies from other sources [19]. Thirdly, the inclusion criteria were limited to studies published in English, which may have introduced language bias and resulted in the omission of relevant studies published in other languages [51]. Fourthly, the review included mainly non-experimental studies, primarily comprising Level III evidence, which may serve to limit the strength of the conclusions drawn [21]. Additionally, most of the included studies were conducted in developed country contexts, which may restrict the generalizability of some of the findings to other, resource-constraint healthcare settings or facilities. Despite these limitations, this review can still provide valuable insights in an underresearched topic such as evidence-informed management. However, researchers should be aware of these limitations and interpret the findings with caution.

### 4.2. Recommendations for Future Research

It is crucial to recognize the current limitations of published evidence in this field. There is a clear necessity for more robust research to improve the validity and generalizability of findings. This would enable the development of evidence-based strategies and interventions to better support nurse managers in effectively utilizing evidence-informed management practices. Therefore, it is recommended that further research be conducted to enhance the validity and breadth of knowledge on evidence-informed management and how its use could be facilitated among nurse managers. Future studies should employ robust research designs, notably large-scale and meticulously crafted randomized controlled trials, spanning various contexts. This is especially pertinent in resource-constrained healthcare environments where novel approaches are needed to promote the adoption of evidence-informed management practices. Such an approach promises to yield a deeper comprehension of the factors shaping the utilization of evidence-informed management by nurse managers and offer insights into strategies for enhancing its utilization and implementation.

Additionally, it is recommended to conduct a systematic review and/or meta-analysis summarizing the impact of evidence-informed management on managerial and patient care outcomes, as these aspects were not the primary focus of the current review. Synthesizing existing evidence in this manner could provide valuable insights into the broader implications of evidence-informed management practices.

Finally, the studies encompassed within this review notably abstained from delineating precise adaptations or modifications in the utilization of evidence-informed management practices by middle managers amid the pandemic. Such a delineation was not explicitly within the purview of this review. However, it warrants consideration whether there exists a discernible divergence or heightened efficacy attributed to the utilization of such practices amid the pandemic's needs. This assessment is particularly pertinent given the pronounced impact of the pandemic on healthcare and nursing management, and expectations for the role within these sectors.

### 4.3. Implications for Nursing Management

This study provides guidance to nurse managers in their work practices. The 13 identified determinants can be considered to enhance nurse managers' utilization of evidence-informed management which has the potential to influence organizational outcomes at the micro-, meso-, and macrolevels, but requires organizational support and health system strengthening. The determinants identified could be effectively integrated into daily management practices through a variety of approaches. One such approach involves benchmarking with other health facilities or organizations to strategically enhance the utilization of evidence-informed management among nurse managers. Additionally, conducting a context analysis can provide insights into which determinants are currently in place and which ones require strengthening to facilitate the seamless integration of evidence-informed management into decision-making processes. This analysis serves as a foundation for developing one or more strategies, complete with defined goals, tasks, timeframes, task prioritization, and clear evaluation criteria [52]. Following the context analysis, the development of strategies may entail the creation of tools, such as educational resources aimed at training nurse managers on evidence-informed management principles, and dashboards designed to streamline the management and analysis of evidence and data [53]. These tools can be adapted from existing resources or developed anew and subsequently incorporated into a comprehensive toolkit to enhance the implementation of strategies prior to pilot testing. Continuous evaluation of the effectiveness of these strategies aimed at optimizing the utilization of evidence-informed management is essential. Feedback loops and the identification of emerging best practices enable the ongoing refinement and adaptation of approaches to ensure their relevance and efficacy in real-world settings [54].

## 5. Conclusions

This comprehensive review identified a total of 13 determinants that significantly influence the utilization of evidence-informed management by nurse managers. These determinants were organized into three overarching themes: (1) Nurse manager determinants in utilization of evidence-informed management at the microlevel, (2) Organizational determinants in utilization of evidence-informed management at the mesolevel, and (3) External stakeholders and context determinants of utilization of evidence-informed management at the macrolevel. The themes were found to be interconnected and interdependent and can be used by nurse managers to optimally utilize evidence-informed management. However, the findings also highlight the need for executive management and policymakers to strengthen health systems by providing resources and support, such as education, incentives, effective communication, robust organizational structures, funding, and ownership. Future studies from various contexts are required to provide a more comprehensive understanding of the determinants influencing nurse managers' utilization of evidence-informed management practices and the use of interventions and strategies to achieve evidence-informed management.

## Figures and Tables

**Table 1:** Determinants for facilitating nurse managers' utilization of evidence-informed management.

Themes	Nurse manager determinants in utilization of evidence-informed management (microlevel) *n* = 5 level of evidence-III(B), V (B)					Organizational determinants in utilization of evidence-informed management (microlevel) *n* = 6 level of evidence-III(B), V(B)						External stakeholders and context determinants of evidence-informed management utilization (macrolevel) *n* = 2 level of evidence-III(B), V(B)	

References (n = 13)	Nurse managers' training, knowledge, skill, and competencies	Nurse manager as knowledge broker	Nurse managers' attitude and behaviour towards evidence-informed management	Nurse managers' experience and level of education	Tools to support evidence-informed management utilization	Robust organizational structures	Adequate and accessible resources	A supportive and learning culture	Information and technology	Positive leadership attitude and behaviours	Ownership and funding structures	External stakeholders	Socioeconomic, political, and ethical context

[27]	x	x	x	x	x	x	x		x		x	x	x
[28]	x	x	x	x	x	x	x	x	x	x		x	x
[29]	x	x		x		x	x	x	x				
[30]	x	x		x	x	x	x	x	x			x	
[1]	x	x	x	x	x	x	x	x	x	x		x	x
[17]	x	x	x	x	x	x	x	x	x	x		x	
[13]	x	x	x	x	x	x	x	x	x	x	x	x	x
[31]	x		x	x		x	x	x	x	x	x	x	x
[32]	x	x	x			x	x	x	x		x	x	x
[2]	x	x	x	x	x	x	x	x	x	x		x	x
[33]	x	x	x			x	x	x	x	x		x	x
[34]	x	x	x	x		x	x	x	x	x		x	
[35]	x	x	x			x	x	x		x			
Total	*n* = 13	*n* = 12	*n* = 11	*n* = 10	*n* = 7	*n* = 13	*n* = 13	*n* = 12	*n* = 12	*n* = 9	*n* = 4	*n* = 11	*n* = 8

**Table 2:** Tools to support evidence-informed management utilization.

Type of tool	Title and source	Description
Models (*n* = 3)	Decision-making dependency model [27]	Eight factors influence the decision-making process: (a) Situation addressed; (b) Time constraints; (c) Input from colleagues; (d) Task and environmental complexity; (e) Decision duration; (f) Resource availability; (g) Decision-making environment; and (h) Personal characteristics
	Evidence-based management model [1, 13, 17]	Understanding evidence-informed management for implementing evidence-based practices in healthcare
	Evidence-based practice model [36]	Focuses on elucidating nurse managers' challenges in implementing evidence-based practice, emphasizing practice change and incorporating research and contextual factors

Decision-making tools (*n* = 1)	Dynamic network analysis decision support tool (DyNADs) [29, 30]	Utilizing computer modelling and simulations to assess the feasibility of innovations within a unit, based on nurse managers' cognitive work analysis. Allows for multilevel, multidimensional testing of interventions, individually or in combination

Framework (*n* = 1)	Framework of facilitators and barriers of evidence-based management [2]	Focuses on identifying facilitators and barriers of evidence-based management

## Data Availability

Data are available on request.

## Supplementary Materials

Supplementary Materials One data extraction table was included. The table outlines the extracted data regarding the reference, aim of the study and country, and findings and implications for practice for each of the 13 articles.
