# Supplementary material for: Facilitating Utilization of Evidence-Informed Management by Nurse Managers in Healthcare Facilities: An Integrative Literature Review
Source: J Nurs Manag. 2024 May 21;2024:6649401. doi: 10.1155/2024/6649401 (PMC11919202; doi:10.1155/2024/6649401)
Supplement: Supplementary Materials — One data extraction table was included. The table outlines the extracted data regarding the reference, aim of the study and country, and findings and implications for practice for each of the 13 articles. [file 6649401.f1.docx]

**Table: Data extraction (n=13)**

| Reference | Aim of the study and Country | Findings | Implication for Practice |
| --- | --- | --- | --- |
| Research Non-experimental Quantitative (n=4) Level III B (Good) | | | |
| Djukic, M., Jun, J., & Fletcher, J. (2021). An Examination of the Factors Associated With Implementation of Evidence‐Based Management Practices for Improving Nurse Work Environments. *Worldviews on Evidence‐Based Nursing*, *18*(2), 129-137. | To study the individual, information and organisational characteristics associated with NMs implementation of the five evidence-based management practices of the Practice Environment Scale of the Nursing Work Index (PES-NWI).  - New York City | - NMs are well positioned and positively influences the staff nurse’s work environments by implementing the five EBMP thereby creating a positive staff culture which leads to improved patient outcomes. - Implementation of actions must be customised to the evidence-based management practice (EBMP) and the context. It is “not a one size fits all”, each practice had unique predictors - Level of education (Bachelor’s versus Associate degree), number of staffs supervised, time spent on research were positively associated with EBMP implementation and alluded to the individual skills of the NM in facilitating EBMP and competing priorities for the NM. - NMs implemented EBMP based on their positive personal experience rather than as a result of stakeholders such as patients, clinical expert or senior leader’s feedback about the practice. - Managers as knowledge brokers would likely decrease the gap between producers and users of the knowledge, thereby bridging the knowledge-practice gap (research-practice gap). - Lack of resources motivated better collegial relationships indicating a positive association and a facilitator of EBMP implementation rather than a barrier. - There is a need to explore organisational cultural attributes as an influencer in manager’s implementation of evidence-based management. | - EIMgt must be tailored to the evidence and context - Managers as Knowledge brokers to use more research information to support EIMgt and bridge the information-practice gap - Individual NM attributes as determinants of EIMgt implementation - Belief in the evidence is important for EIMgt implementation - Organisation’s cultural attributes as an influencer of EBMgt |
| Hasanpoor, E., Siraneh Belete, Y., Janati, A., Hajebrahimi, S., & Haghgoshayie, E. (2019a). Nursing Managers’ Perspectives on the Facilitators and Barriers to Implementation of Evidence‐Based Management. *Worldviews on Evidence‐Based Nursing*, *16*(4), 255-262. | A descriptive cross-sectional study design using nursing managers across 20 hospitals in Iran to evaluate NM’s perspectives on bariers and facilitators to using in evidence-based management (EBMgt) | - Significant barriers to evidence-based management included: nursing managers’ expertise and skills, domain of Training and research systems, communication between knowledge producers and hospital decision-makers (research-practice gap) - Facilitators identified included: social/interpersonal factors, positive response for scientific management principles - Barriers to use of evidence unique to low-middle income countries is lack of innovation, incentives and resources when compared to high income countries such as Australia, where lack of time is the main perceived barrier. - Other barriers included: lack of time, lack of incentives, information that were not significant, difficulty recognizing, lack of NM skills related to searching, critically appraising and interpreting and applying study findings to change management practice lack of empowerment - Recommendation: Utilise education of EBMgt application of management practice as an empowering strategy to improve management practice. - Organisational support, adequate resources and continuing education was cited as important facilitators of EBMgt | - Barriers to EBMgt - Facilitators to using EBMgt - Gap between evidence production and evidence utilisation - Research-practice gap - Context (Country and organisational), resources, education, attitudes of nurse managers. |
| Hasanpoor, E., Belete, Y. S., Janati, A., Hajebrahimi, S., & Haghgoshayie, E. (2019b). The use of evidence-based management in nursing management. *Africa Journal of Nursing and Midwifery*, *9*(21), 1. | A descriptive cross-sectional study design using nursing managers across 20 hospitals in Iran to evaluate the use of information and identify the chief information sources used in evidence-based management (EBM) | - Types of managers identified: Scientific oriented, data and fact-based managers, plan-based managers, expertise oriented, ethics oriented, customer-oriented managers. - Majority of the managers used personal experience for decision-making - The selection of the best evidences and sources of evidences are driven by the problem and the context and people it is affecting. - Stakeholder values, key capabilities, skills, knowledge, evidence sources and benefits should be considered. - Training managers for skill in use of evidence facilitates use of information | - Sources of information - Types of managers based on utilisation of information - Training NMs is a facilitator of using EIMgt |

| Hilal, N., Harb, S., Jamal, D., & El‐Jardali, F. (2020). The use of evidence in decision making by hospital managers in Lebanon: A cross‐sectional study. *The International Journal of Health Planning and Management*, *35*(1), e45-e55. | A cross sectional web survey design used to evaluate the level to which middle and senior hospital managers in Lebanon use evidence in their decision-making practices and to identify barriers to and facilitators for the use of information | - High responses received to evidence-seeking behaviours and high use of evidence - Timeliness of information received and irrelevant information were not perceived as barriers in this study setting - Demographics of the study showed younger managers who due to recency of training, may have received education on use of evidence in their training - Facilitators identified to use of evidence in decision-making included: incentives for knowledge use or production, administrative structure supportive of evidence-based decision-making, evidence utilisation in decision-making is requested by senior leaders, available resources and access to health research, formal and informal networks with researchers available offering support in using evidence is available. Presence of a research culture - Barriers: lack of funding, training - The managers agreed that training was needed on integrating evidence in decision-making, finding and using research. - Funding and budget for conducting research and primary research being conducted in the hospital was varied - Resources identified: web support, evidence-based management unit, networking with researchers external to the facility, funding, training on use of evidence - Packaged evidence was more useful - Managers used evidence to formulate policies, protocols, procedures, seeking solutions to nursing issues, purchasing decisions - Gaps in the research: determine the quality of evidence that facilities include in their decisions | - Context (Low-middle income countries) and Eastern Mediterranean Countries (EMR) and EBMgt - Education and Training on seeking evidence, integrating evidence into decision-making - Hospital managers as producers of research - Barriers - Facilitators - Capacity of NMs |
| --- | --- | --- | --- |
| Research Non-experimental Qualitative (n=6) Level III | | | |
| Chisengantambu-Winters, C., Robinson, G. M., & Evans, N. (2020). Developing a decision-making dependency (DMD) model for nurse managers. *Heliyon*, *6*(1), e03128. | To determine the types of decisions made by NMs and the related decision-making processes. The study also proposed a dependency model for decision-making.  - South Australia | - Types of decisions made: Managerial (operations), Clinical (Patient care), Executive (Strategic). - Decisions are influenced by context, timeframe required to make the decision, the problem (how it is presented, who is presenting it), why is a decision required, stakeholders involved and affected by the decision, the anticipated outcome (Who, When, why, What, How -4W +1H) - Dependency factors of decision-making: (Multiplicity of NM role, timeframe available to make decision, duration of decision-making, complexity of the decision, consultation with others, environment, resources available, personal characteristics of the decision-maker) - Increasing uptake of the model requires awareness, education and training, disseminating, implementation, evaluation - Nine steps in the Dependency model, sequential, scaffolding steps, to improve decision-making - Only some NMs followed a clearly distinguishable pattern or process of decision-making | - Types of decisions made - Knowledge, skills and experience of the NMs - Decision-making processes - Decision-making/support Dependency tools and models - Contextualise decisions - context matters |
| Effken, J. A., Brewer, B. B., Logue, M. D., Gephart, S. M., & Verran, J. A. (2011). Using cognitive work analysis to fit decision support tools to nurse managers’ work flow. *International journal of medical informatics*, *80*(10), 698-707. | A qualitative descriptive study design based in Arizona to conduct a Cognitive Work Analysis (CWA) and Work Domain Analysis (WDA) of NMs that will lead to the development and implementation of a decision support tool. | - Influence of NMs work environment and domain on need for decision support tools - Constraints from NM domain: large volumes of data available to NMs that require synthesising in order to make decisions, majority of their time is spent on facilitating efficient patient flow, ensuring patient satisfaction, time spent on monitoring and implementing patient safety and quality initiatives, multiple initiatives ongoing at the same time, multiple data sources, validation of data, limited time for planning - Recommendation: decision support tool that collates all activities and data, prioritises them and test likelihood of various strategies that may lead to a favourable outcome, allowing proactive thinking rather than retrospective reactions - NMs collected information systematically, however they reached conclusions quickly often with education as a remedial action - NMs found dashboards with summarised information helpful as it retrieved information from multiple sources and displayed them in a collated and summarised format - Integration of IT solutions detracted from patient care assessment priorities - NMs responsible for large complements of staff and their time is taken up with various patient safety and quality initiatives. - Staff on unit have varied competencies - NM decisions driven by external stakeholders e.g. Joint Commission | - NMs work environment influences decision - Competing priorities for NMs - NM capacity in making decisions - Simulated decision support tools for NMs - Technology as a distracter - Integration of multiple sources of information into dashboards |
| Effken, J. A., Verran, J. A., & Logue, M. D. (2010). Nurse managers' decisions: fast and favoring remediation. *The Journal of nursing administration*, *40*(4), 188. | A qualitative descriptive study design based in Arizona to determine how NMs make decisions and that will lead to the development and implementation of a decision support tool. | - Require decision-support tools that prospectively evaluate the outcome of a decision (Created the Computerised DyNADS tool) - Must understand the process used by NMs to make decisions for the tool to be effective – high cognitive workload burdening NMs - Topographic and Symptomatic categories of search strategies: Topographic (access to how the process works in memory or diagram) Symptomatic (pattern recognition, decision-support diagrams, testing). - Nearly all managers followed the decision ladder, only 2 of the10 followed 7 or more of the 8 steps, 9 jumped from alert to solution. - Education frequently selected action for staff remediation - Search strategy was decision table with limited ‘what then’ options - NMs skipping steps may be related to experience, experience is measure not only in years of experience but related to frequency the NM was exposed to or experienced that problem or situation. - NMs did not mention performance criteria when selecting the decisions, this may lead to sub-optimal outcomes | - NMs decision-making is influenced by organisational culture and leadership support - NM experience related to the problem - Symptomatic versus topographical NM category - Cognitive processing ability of the NM - Computerised Decision- support tools - Real time or prospective decision-support tools - Cognition |
| Spiers, J. A., Lo, E., Hofmeyer, A., & Cummings, G. G. (2016). Nurse leaders' perceptions of influence of organizational restructuring on evidence-informed decision-making. *Nurs Leadersh*, *29*, 64-81. | A qualitative descriptive study based in West Canada aimed to describe how organisational context and restructuring influenced nurse leader’s use of evidence in decision-making in their management practice. | - Search for information and it’s source is driven by the type of decision to be made - Sources included informal sources of information for complex matters that required value judgment and non-clinical matters and formal sources e.g. written documents, policies, procedures, university academic sites, clinical reference groups, professional organisations - Understanding the issues were important prior to searching for information - Information was customised to suit the context and issue - Facilitators: good strategic relationships were important for communication and consultation with stakeholders e.g. with staff - Context is dynamic, needs adaptation and agility - Decisional inertia as a result of constant change, lack of communication and transparency, bureaucratic hierarchies leading to delays in decisions and system inefficiencies - Frustration from lack of autonomy, empowerment, transparency and communication of information in the change process - Increasing workloads - EIDM is a social process, value laden process, politically and contextually influenced - Having the strategic direction in line of vision and the big picture helps in times of change to keep the focus. | - Sources of information - Drivers of information sources - Context is complex, dynamic and requires customisation - Effective communication and knowledge sharing is important - Networking with stakeholders - Lack of empowerment and autonomy - Workloads and role responsibilities - Definition of EIMgt (value laden and social process) |
| Urquhart, R., Kendell, C., Folkes, A., Reiman, T., Grunfeld, E., & Porter, G. A. (2018). Making it happen: middle Managers' roles in innovation implementation in health care. *Worldviews on Evidence‐Based Nursing*, *15*(6), 414-423. | A qualitative study design based in Canada, to study middle manager’s role in implementing innovation and their experience of the implementation. | - Managers’ role in implementing new Innovation, tools, instruments, technology after the decision is made includes that of planner, coordinator, facilitator, motivator, and evaluator - Manager’s decision-making power limited by senior leaders’ confines - The process of implementing new innovation from managers perspective was seen as collaborative - Implementing innovation was viewed as additional responsibility, that they were not trained formally and prepared for, lack of decision-making authority on ‘big matters’ - Managers’ role is crucial and goes beyond closing information gaps and acting as knowledge brokers - Decreased uptake of innovation has more to do with failure of implementation than dissemination. Implementation more important than adoption - Not clearly defined roles of the manager in healthcare, managers may be responsible for clinical care and management leading o role confusion and being unprepared - Managers to participate actively in decision-making rather than only providing the information for senior leaders to make the decision - Effectiveness of implementation must be evaluated | - Managers as implementers - Managers as knowledge brokers - Training - Lack of empowerment, authority - Implementation is more important than adoption and dissemination of innovation - Preparation for role of manager - Evaluation of implementation outcomes - Building NM capacity to implement innovation |
| Wilkinson, J. E., Nutley, S. M., & Davies, H. T. (2011). An exploration of the roles of nurse managers in evidence‐based practice implementation. Worldviews on Evidence‐Based Nursing, 8(4), 236-246. | A qualitative study design using a case study approach was employed to study the role NMs play in evidence-based practice implementation in acute health care settings across four Scottish health care boards. | - NMs roles and responsibilities in facilitating EBPI   - Act as leaders and champion EBPI, active participation   - Empower nurses - EBPI is dependent on contextual factors - Visible leadership support - NMs as linkages to clinical governance structures within the unit to support EBPI - NMs need to understand the process of EBPI - NMs attitudes and behaviours influence EBPI, positive attitudes increased uptake of EBPI - NMs need to have the knowledge and skills to understand the EBPI process and facilitate its implementation | - Context - Organisational and Leadership support - NMs role as facilitators and champions of EBPI |
| Research Non-experimental Mixed Methods (n=1) Level III | | | |
| Ivankovic, D., Poldrugovac, M., Garel, P., Klazinga, N. S., & Kringos, D. S. (2020). Why, what and how do European healthcare managers use performance data? Results of a survey and workshop among members of the European Hospital and Healthcare Federation. *PloS one*, *15*(4), e0231345. | A descriptive cross-sectional survey study design based in Europe exploring the use of performance management data in health care facilities. | - Survey and interactive workshop implemented - Survey: Use of performance data and how it can be enhanced - **Why is performance data used**: decision-making is supported by evidence, evidence based on performance data helps easily justify decision-making,   - Strategic managers use evidence more for justification and clinical managers or process support managers use performance data more to inform decisions.   - Internal and external motivation to use performance data includes internal quality assurance and improvement and accountability and benchmarking respectively.   - Benchmarking valuable to identify best practices and strategies used to perform better.   - Privately owned facilities use more performance data than public facilities   - More confidence in performance data required in order for managers to use it more. - **What performance data:** Large quantities of data is collected, but little is reported or used, soft intelligence is missing to action and inform decisions, patient data reporting lags behind. - Data collection sources include: administrative data, data from electronic medical record (EMR) available. Require more patient reported data and reported through a single EMR. - **How is performance data reported**: written reports, dashboards, control charts, verbal reporting and scorecards - Confidence in data skills and competencies lacking, require training - Lack of time to analyse data and integrate it into decisions - **Reflective workshop** | - Use of organisational data (performance data) - Context drives the need for information - Confidence in the reliability and validity of data - Soft intelligence required to action data - Sources of performance data - Format of performance data - Capacity building for use of performance data, skills, knowledge needed |

| **Non-research: Literature reviews (n= 2) Level V Evidence** | | | |
| --- | --- | --- | --- |
| Hasanpoor, E., Hajebrahimi, S., Janati, A., Abedini, Z., & Haghgoshayie, E. (2018b). Barriers, facilitators, process and sources of evidence for evidence-based management among health care managers: a qualitative systematic review. *Ethiopian journal of health sciences*, *28*(5). | A literature review conducted to review the factors and components of Evidence-based management (EBMgt) and propose a framework for EBMgt | - Managers have positive attitudes to using evidence, however they primarily use personal experience as information source - Barriers: Training and research systems, lack of communication between knowledge brokers (KB) and knowledge producers (KP) - Facilitators: Social and interpersonal factors, willingness to use scientific evidence in management - Barriers and facilitators were indicative of LMIC as opposed to high income countries showing lack of time as the main barrier. - Determinants of EIMgt are context driven e.g. in the US setting: workforce leadership, organizational climate and culture, relationships and partnerships, and financial processes - Competencies and skills in searching, appraising, applying information into management practice are required - Use multiple sources of evidence - Development of the framework for EBMgt should be based on facilitators, barriers, sources of evidence, and process of EBMgt decision making. - EBMgt is continuous | - Determinants of EIMgt: Barriers, Facilitators - Determinants of EIMgt are context driven, interrelated and dynamic (local, national and global contexts) - Determinants are interrelated - Sources of evidence - Managers as knowledge Brokers and Knowledge Producers - Bridge the gap between KB and KP - Education and training for managers in EBMgt use - EBMgt training must be formalised - Organisational infrastructure is important - Elements /definition of EBMgt |
| Shafaghat, T., Imani Nasab, M. H., Bahrami, M. A., Kavosi, Z., Roozrokh Arshadi Montazer, M., Rahimi Zarchi, M. K., & Bastani, P. (2021). A mapping of facilitators and barriers to evidence-based management in health systems: a scoping review study. *Systematic reviews*, *10*(1), 1-14. | A scoping review conducted to identify the potential facilitators and barriers using evidence-based management in health systems | - Facilitators and barriers: 6 main aspects: Attitude towards research, External factors, contextual factors, policies and procedures, resources, research capacity and data availability. - Other reported facilitators and barriers grouped into: informational, organizational, individual, and interactional. | - Facilitators and barriers |
